# A Filtering Algorithm of MEMS Gyroscope to Resist Acoustic Interference

**DOI:** 10.3390/s20247352

**Published:** 2020-12-21

**Authors:** Yufei Sun, Peng Guo, Lihui Feng, Chaoyang Xing, Junjie Wu

**Affiliations:** 1 The Key Laboratory of Photonics Information Technology, Ministry of Industry and Information Technology, School of Optics and Photonics, Beijing Institute of Technology, Beijing 100086, China; 3120180566@bit.edu.cn (Y.S.); lihui.feng@bit.edu.cn (L.F.); 3120190583@bit.edu.cn (J.W.); 2 Beijing Institute of Aerospace Control Device, Beijing 100854, China; mems13@163.com

**Keywords:** orthogonal demodulation, acoustic interference, MEMS gyroscope

## Abstract

To reduce the impact of acoustic interference in a microelectromechanical system (MEMS) gyroscope and to improve the reliability of output data, a filtering algorithm based on orthogonal demodulation is proposed. According to the working principle and failure mechanism of a MEMS gyroscope, the sound and angular velocity frequencies are not identical, which lead to a different frequency signal output of the original single-channel demodulation scheme. Therefore, a Q channel demodulation filtering process was added to the origin single-channel demodulation scheme. For the Q channel demodulated signal, a Hilbert transform was used to compensate for the 90 degree phase shift. The IQ dual-channel difference can remove the acoustic interference signal. The simulation results indicate that the scheme can effectively suppress the acoustic interference signal and it can eliminate more than 95% of the impact of sound waves. We assembled the acoustic interference experimental platform, collected the driving and sensing data, and verified the denoising performance with our algorithm, which eliminated more than 70% of the noise signal. The simulation and experimental results demonstrate that the scheme can eliminate acoustic interference signal without destroying angular velocity signal.

## 1. Introduction

A microelectromechanical system (MEMS) gyroscope is a type of sensor that is used to measure angular velocity. It is widely used because it is small in size, lightweight, low cost, and has low power consumption [1,2,3,4]. It has a fixed resonant frequency, specifically, the driving frequency in the direction of the driving shaft and the sensing frequency in the direction of the sensing shaft. When the acoustic frequency is close to the natural frequency of the gyroscope, resonance occurs. If the sound pressure level is high, stronger interference occurs. The demodulation system outputs an incorrect angular velocity signal after coherent demodulation and low-pass filtering when disturbed by acoustic interference [5,6,7,8]. Therefore, it is necessary to take measures to suppress the acoustic interference effect.

The main protection measures include adding protective devices and algorithm protection. In terms of protective devices, the effect of acoustic interference can be reduced by coating the gyroscope shell with a layer of sound absorption material, and a damping shell can also be added. Hardware protection increases the volume of the sensor, rendering the sensor inconvenient to use, and usually only has a protective effect on the partial frequency band of sound interference. Pregassen S. et al. [9,10] proposed a noise reduction method that involved coating MEMS gyroscopes with sound-absorbing material. An acoustic test on a MEMS gyroscope wrapped with sound absorption material indicated that the noise interference could be reduced by 90%. This type of sound absorption material is only effective for a specific frequency band of sound waves, therefore, it needs to be prepared and designed according to the resonant frequency of the gyroscope. By improving the structure of the MEMS gyroscope and increasing its natural frequency, it can differ greatly from certain high-frequency bands of acoustic waves [11], however, when the noise frequency also rises, this method does not work. This solution does not reduce the impact of sound from the source. The gyroscope with double mass blocks, also fails to deal with the interference effect of sound waves, because of the difference in the sound field distribution between the two mass blocks and the defects in the manufacturing process [12,13].

In terms of algorithm protection, filtering schemes to suppress noise have been researched. The common analysis methods have been Kalman filtering, particle filtering, wavelet denoising, neural networks, and so on [14,15,16,17,18,19,20]. A more complex solution may not lead to better filtering performance. The specific method should be adopted according to the characteristics of the noise received by the MEMS gyroscope. For example, Diao et al. proposed a method that combined Allan variance, time series analysis, and a Kalman filter to compensate for the drift in a MEMS gyroscope [15]. They built an autoregressive model and an autoregressive moving average model of gyroscope drift. Afterwards, the Kalman filter was designed to reduce the random error of the gyro. Cho S.Y. et al. investigated a fusion filter [21]. The IIR filter and FIR filter were processed separately. Then, the two filters were combined, according to the mixing probability calculated by the specific parameters of the two filters, and then mainly used to solve the issues relating to model uncertainty and temperature random noise. Zhang et al. put forward a new UFCL (using former period characteristics to compensate latter smoothness data) method for the compensation of the MEMS gyroscope zero drift [22]. The periodic error could be compensated in real time. It can be seen that the above schemes were aimed at reducing the noise and random deviation of the gyroscope during use to ensure a reliable output.

At the same time, there are more and more ways to attack MEMS gyroscopes. The acoustic interference mentioned above is a relatively new attack method, and relevant researchers have been carrying out corresponding research on the attack principle and protection measures for MEMS gyroscopes [5,6,7,8,9,10,11,12,13]. In this paper, using a software protection method through analyzing the working principle and failure mechanism of the gyroscope, we propose a noise reduction scheme based on IQ dual-channel quadrature demodulation filtering algorithm. In order to demodulate the angular velocity signal and to reduce the influence of noise, a Hilbert transform was used to compensate for the 90 degree phase shift after Q channel demodulation. This provided an algorithm design for the development of an anti-interference MEMS gyroscope.

The structure of the remainder of the article is as follows: In Section 2, we analyze the working principle and failure mechanism of a MEMS gyroscope using dynamic equations; in Section 3, we show our filtering algorithm based on orthogonal demodulation; in Section 4 and Section 5, we present the theoretical simulation results based on the dynamic equations and the output results of the algorithm with the collected data as the input; and in Section 6, we explain some details of the scheme and also propose an acoustic warning scheme based on orthogonal demodulation.

## 2. Motion Analysis of MEMS Gyroscope under Acoustic Interference

For a MEMS gyroscope, in the direction of the driving comb, the electrostatic force is used as the driving force for the mass block to vibrate with constant frequency (driving frequency) along the driving direction. When the angular velocity in the direction perpendicular to the driving direction and the sensing direction is applied, due to the Coriolis effect, a Coriolis force in the sensing direction is generated which is proportional to the angular velocity to be measured. The displacement of the mass block in the sensing direction can be obtained by the change in the capacitance. The displacement is amplified, coherently demodulated, and low pass filtered, and the angular velocity signal is recovered.

When there is acoustic interference, the effect of sound can be equivalent to that of force. The motion of a MEMS gyroscope can be described by a dynamic equation [9,23,24,25,26] as follows:

(1) $$\begin{array}{l} \left[\begin{array}{cc} m_{x} & 0\\ 0 & m_{y} \end{array}\right]\left[\begin{array}{c} \ddot{x}\\ \ddot{y} \end{array}\right]+\left[\begin{array}{cc} D_{xx} & D_{xy}\\ D_{yx} & D_{yy} \end{array}\right]\left[\begin{array}{c} \dot{x}\\ \dot{y} \end{array}\right]+\left[\begin{array}{cc} k_{xx} & k_{xy}\\ k_{yx} & k_{yy} \end{array}\right]\left[\begin{array}{c} x\\ y \end{array}\right]\\ \;=\left[\begin{array}{cc} 0 & 2\Omega (t)m_{y}\\ -2\Omega (t)m_{x} & 0 \end{array}\right]\left[\begin{array}{c} \dot{x}\\ \dot{y} \end{array}\right]+\left[\begin{array}{c} F_{X}\\ F_{Y} \end{array}\right]+\left[\begin{array}{c} F_{Nx}\sin(\omega_{n}t+\varphi_{Nx})\\ F_{Ny}\sin(\omega_{n}t+\varphi_{Ny}) \end{array}\right] \end{array}$$

where x and y are the driving and sensing displacements, respectively; $\dot{x}$ and $\dot{y}$, $\ddot{x}$ and $\ddot{y}$ are the velocity and acceleration in the driving and sensing directions, respectively. $m_{x}$, $m_{y}$ are the driving and sensing masses, respectively; $D_{xx}$ and $D_{yy}$, $k_{xx}$ and $k_{yy}$ are the damping and elastic coefficients in the driving and sensing directions, respectively. $D_{xy}$, $D_{yx}$, $k_{xy}$, $k_{yx}$ are the non-proportional damping and non-equal elasticity, which represent the interactions of motion in the driving and sensing directions, respectively. Ω(t) is the angular velocity to be measured; $F_{X}$ and $F_{Y}$ are the forces in the driving and sensing directions without acoustic interference; $F_{X}$ can be expressed as $F_{X}=F_{x}\sin(\omega_{d}t)$, where $F_{x}$ is the magnitude of the driving force and $\omega_{d}$ is the driving frequency. $F_{Nx}$ and $F_{Ny}$ are the sound pressure acting on the driving and sensing directions, respectively; $\omega_{n}$ is the sound frequency; $\varphi_{Nx}$ and $\varphi_{Ny}$ are the sound phases in the driving and sensing directions, respectively.

In order to analyze the displacement signal of a gyroscope under acoustic pressure and facilitate the calculation process, we simplify Equation (1). There is no external force in the sensing direction except for acoustic interference, that is, $F_{Y}=0\;N$. The movement in the sensing direction is very small. The coupling coefficient between the driving and sensing directions is also a small value. Thus, the influence of the sensing mode coupling to the driving mode can be ignored, namely, the influence of $D_{xy}$ and $k_{xy}$ in Equation (1) can be removed. However, because of the limitations in the manufacturing process, the influence of the motion coupling from the driving to the sensing direction is relatively large, which cannot be ignored. Finally, by solving the simplified dynamic equation, we can obtain the following:

(2) $$\begin{array}{l} y_{N}(t)=2A_{x}A_{y}F_{x}\Omega (t)m_{x}\omega_{d}\cos(\omega_{d}t+\varphi_{x}+\varphi_{y})\\ \;+A_{x}A_{y}F_{x}D_{yx}\omega_{d}\cos(\omega_{d}t+\varphi_{x}+\varphi_{y})\\ \;+A_{x}A_{y}F_{x}k_{yx}\sin(\omega_{d}t+\varphi_{x}+\varphi_{y})\\ \;+2A_{Nx}A_{Ny}F_{Nx}\Omega (t)m_{x}\omega_{n}\cos(\omega_{n}t+\varphi_{Nx}+\varphi_{Nxx}+\varphi_{Nxy})\\ \;+A_{Nx}A_{Ny}F_{Nx}D_{yx}\omega_{n}\cos(\omega_{n}t+\varphi_{Nx}+\varphi_{Nxx}+\varphi_{Nxy})\\ \;+A_{Nx}A_{Ny}F_{Ny}k_{yx}\sin(\omega_{n}t+\varphi_{Nx}+\varphi_{Nxx}+\varphi_{Nxy})\\ \;-A_{Ny}F_{Ny}\cos(\omega_{n}t+\varphi_{Ny}+\varphi_{Nyy}) \end{array}$$

where $A_{x}={\left[\sqrt{{\left(k_{xx}-m_{x}\omega_{d}^{2}\right)}^{2}+{\left(D_{xx}\omega_{d}\right)}^{2}}\right]}^{-1}$ and $A_{y}={\left[\sqrt{{\left(k_{yy}-m_{y}\omega_{d}^{2}\right)}^{2}+{\left(D_{yy}\omega_{d}\right)}^{2}}\right]}^{-1}$ represent the amplitude frequency characteristics of the driving and sensing directions, respectively. $\varphi_{x}=\arctan(\frac{k_{xx}-m_{x}\omega_{d}^{2}}{D_{xx}\omega_{d}})$ and $\varphi_{y}=\arctan(\frac{k_{xx}-m_{x}\omega_{d}^{2}}{D_{xx}\omega_{d}})+\arctan(\frac{k_{yy}-m_{y}\omega_{d}^{2}}{D_{yy}\omega_{d}})$ represent the phase frequency characteristics of the driving and sensing directions, respectively.

$A_{Nx}={\left[\sqrt{{\left(k_{xx}-m_{x}\omega_{n}^{2}\right)}^{2}+{\left(D_{xx}\omega_{n}\right)}^{2}}\right]}^{-1}$ represents the amplitude frequency characteristics of the displacement interference signal in the driving direction; $\varphi_{Nxx}=\arctan(\frac{k_{xx}-m_{x}\omega_{n}^{2}}{D_{xx}\omega_{n}})$ represents the phase frequency characteristics of the displacement interference signal in the driving direction. $A_{Ny}={\left[\sqrt{{\left(k_{yy}-m_{y}\omega_{n}^{2}\right)}^{2}+{\left(D_{yy}\omega_{n}\right)}^{2}}\right]}^{-1}$ represents the amplitude frequency characteristics of the displacement interference signal in the sensing direction. $\varphi_{Nyy}=\arctan(\frac{k_{yy}-m_{y}\omega_{n}^{2}}{D_{yy}\omega_{n}})$ and $\varphi_{Nxy}=\varphi_{Nxx}+\varphi_{Nyy}$ represent the phase frequency characteristics of displacement interference signal in the sensing direction.

The first three terms of Equation (2) are the displacement signals in the sensing direction without acoustic interference, and the last four terms are the displacement interference signal caused by acoustic waves. According to the interference sources, the four displacement interference signals can be divided into two parts. The first part consists of four to six terms, which are caused by the displacement interference signals in the driving direction coupled to the sensing direction through the Coriolis force, non-proportional damping, and unequal elasticity. The second part is the seventh item in the equation, which is the displacement interference signal generated by the direct action of acoustic waves on the sensing direction.

Because the effect of the acoustic interference on the driving direction coupled to the sensing direction is relatively small, when the acoustic interference acts on the sensing direction, it will have a greater impact.

## 3. Filtering Algorithm Based on Orthogonal Demodulation

Generally speaking, the closed-loop control algorithm in the driving direction can ensure that the gyroscope vibrates with constant frequency and amplitude. When acoustic interference is present in the working environment of the gyroscope, the acoustic wave only has a short-term impact on the driving displacement, and then it is immediately corrected by the closed-loop control algorithm. It can be seen that the closed-loop control algorithm can eliminate the displacement interference signal in the driving direction, namely the four to six terms in Equation (2), outlined above. Therefore, the noise elimination scheme we propose is mainly used to suppress the displacement signal that is caused by the acoustic wave directly acting on the sensing direction.

It was found that the angular velocity signal was an amplitude modulation signal of the bilateral band, while it is hard to achieve this with acoustic waves generated intentionally. It is more common that the acoustic frequency is close to the driving frequency, but not identical. According to the difference between the acoustic and angular velocity signals, an anti-acoustic interference scheme based on orthogonal demodulation is proposed. The angular velocity signal with high-frequency sampling is demodulated using two-way orthogonal demodulation, and the two-way differential is used to eliminate the single side-band acoustic directional attack.

**Figure 1:**
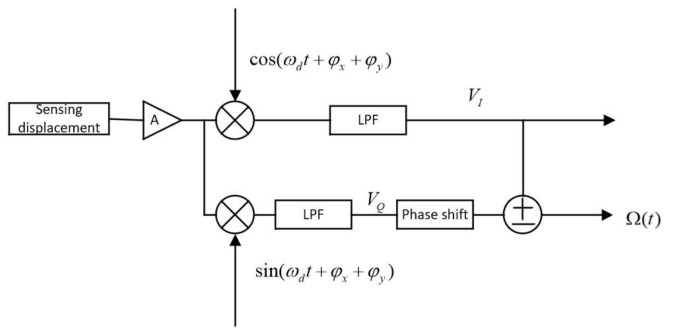
Structure of the filtering algorithm based on IQ dual-channel orthogonal demodulation.

The structure of the filtering algorithm is based on IQ dual-channel orthogonal demodulation, as shown in Figure 1. The sensing displacement obtained by calculating the dynamic equation, which is expressed in Equation (2), is the input of the scheme. The original I channel demodulation process includes coherent demodulation and low-pass filtering. The demodulation signal frequency is equal to the driving frequency, and the phase is equal to that of the sensing signal. In the process of Q channel demodulation, the orthogonal demodulation signal, which has a phase difference of 90° to that of the driving signal is used to demodulate the sensing displacement signal. The frequency of the orthogonal demodulation signal is also the driving frequency. After low-pass filtering, the Hilbert transform is used to compensate for the 90° phase shift. Finally, the two signals are added and subtracted. 

In the presence of acoustic interference, each step of the proposed scheme is analyzed. The demodulated outputs of channel I and channel Q can be expressed as follows:

(3) $$V_{I}=\Omega (t)+\frac{D_{yx}}{2m_{x}}+\frac{F_{Ny}A_{Ny}}{2}\cos(\omega_{n}t-\omega_{d}t+\varphi_{Nyy}-\varphi_{x}-\varphi_{y})$$

(4) $$V_{Q}=\frac{k_{yx}}{2m_{x}\omega_{d}}-\frac{F_{Ny}A_{Ny}}{2}\sin(\omega_{n}t-\omega_{d}t+\varphi_{Nyy}-\varphi_{x}-\varphi_{y})$$

The Q channel demodulation signal is orthogonal to the I channel demodulation and angular velocity signals. The multiplication of the quadrature signal produces a double-frequency quantity, and then the high-frequency component is filtered out through low-pass filtering, which includes the angular velocity signal, i.e., the component caused by non-proportional damping coefficient $D_{yx}$. Similarly, in the I channel, the component caused by the unequal elastic coefficient $k_{yx}$ is also be removed after low-pass filtering. It can be seen in Equations (3) and (4) that the frequency of the interference signal caused by the sound wave is equal, but the phase difference is 90 degrees, which can be compensated by the Hilbert transform. After the Hilbert transform, the direct current caused by $k_{yx}$ in the Q channel signal is also eliminated. Thus, it can be inferred from Equation (4) that the output signal of the Q channel only contains the interference signal caused by sound. When the acoustic frequency is less than the driving frequency, the Q channel signal is 90° ahead because of the Hilbert transform, and the displacement interference signal can be filtered by adding the two IQ signals. When the acoustic frequency is greater than the driving frequency, the Q channel signal lags by 90° and the displacement interference signal can be filtered by subtracting the two IQ signals. According to the expression of Equations (3) and (4), the IQ dual-channel quadrature demodulation filtering algorithm can ensure the integrity of the angular velocity signal, and also filter out the displacement interference signal.

## 4. Simulation Results

By solving the dynamic equations of the MEMS gyroscope in the acoustic environment, we can obtain the sensing displacement data, which are imported into the algorithm as the input. The output signal of single-channel demodulation and that of IQ dual-channel orthogonal demodulation were compared and analyzed. To realize the filtering performance and avoid the problem of time-domain signal delay caused by the high-order filter, we adopted a second-order IIR filter, with a cutoff frequency of 10 Hz. The simulation parameters were set as in Table 1 [13].

**Figure 2:**
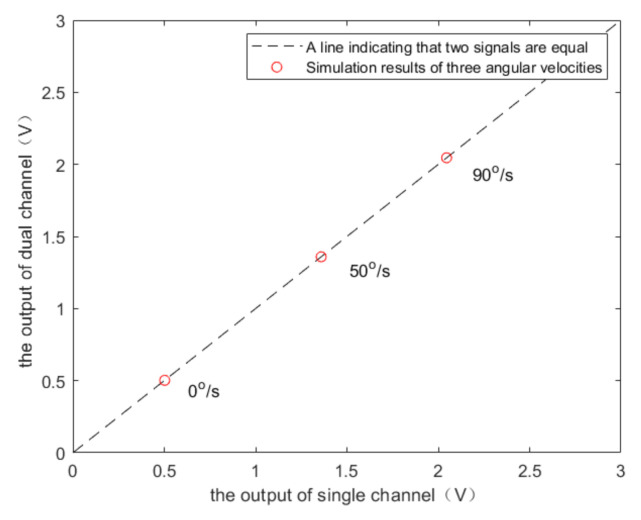
Simulation results of the microelectromechanical system (MEMS) gyroscope output signal in the absence of acoustic interference.

The output angular velocity signal can be obtained after demodulation. In the absence of acoustic interference, the results of single-channel demodulation and dual-channel demodulation are shown in Figure 2. It can be seen from the simulation results that the output signals of the two demodulations are the same. The proposed scheme does not destroy the integrity of the angular velocity signal.

**Figure 3:**
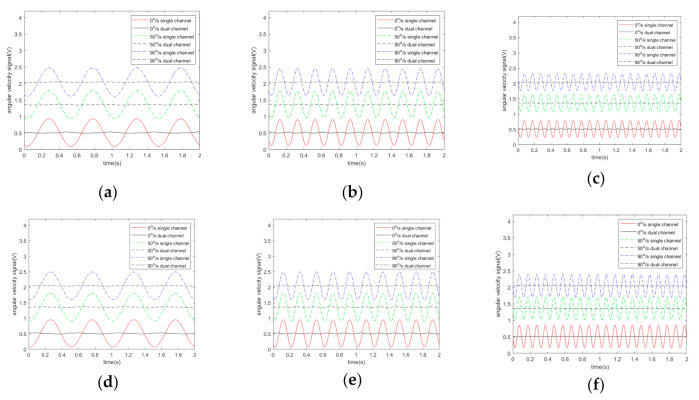
Simulation results of MEMS gyroscope output signal at different acoustic frequency. (**a**) 5600 Hz; (**b**) 5597 Hz; (**c**) 5592 Hz; (**d**) 5604 Hz; (**e**) 5607 Hz; (**f**) 5612 Hz.

In the presence of acoustic interference, the corresponding results are shown in Figure 3, which reveal the outputs of the original single-channel and IQ dual-channel quadrature demodulations. Figure 3a–f shows the effect of acoustic interference under six different sound frequencies. The interval between sound frequency and driving frequency was set as 2, 5, and 10 Hz. The output of demodulation is displayed in the figure when the sound frequency is both greater and less than the driving frequency.

**Figure 4:**
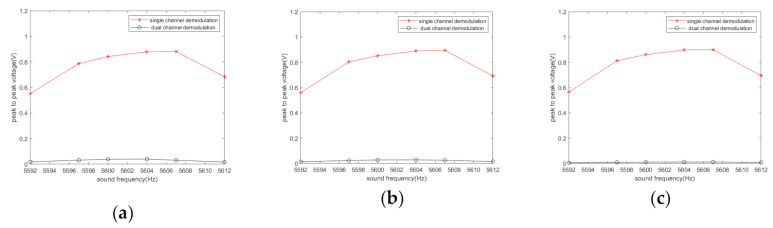
Simulation results of the peak-to-peak values of output signals at different angular velocities. (**a**) 0°/s; (**b**) 50°/s; (**c**) 90°/s.

It can be seen from Figure 2 and Figure 3 that when the angular velocity is 0°/s, the demodulation output is not 0 V, which is due to the influence of the non-proportional damping coefficient $D_{yx}$. At different angular velocities, the peak-to-peak values of output signals of single-channel demodulation and dual-channel demodulation are shown in Figure 4.

**Figure 5:**
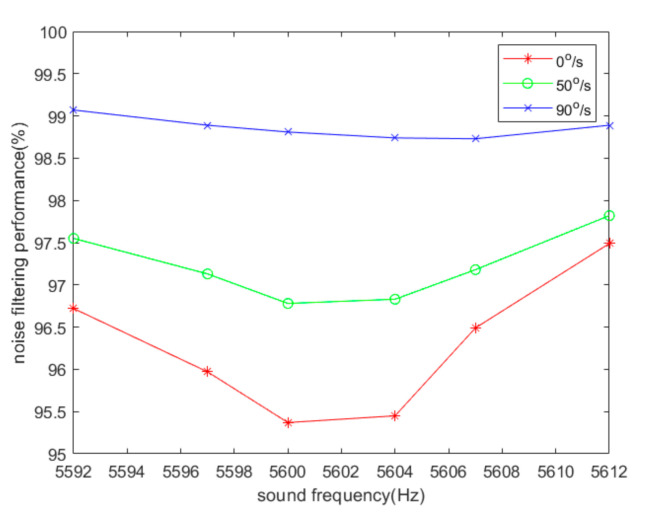
Noise-filtering performance of the MEMS gyroscope based on simulation.

Figure 3 andFigure 4 indicate that sound affects the output of the demodulation, the amplitude and frequency of which depend on the difference between the acoustic frequency and the driving frequency. Figure 4 shows that the angular velocity of the gyroscope does not have a great influence on the acoustic interference. If the sound frequency is closer to the driving frequency, the frequency of the output signal of the single-channel system is lower and the interference is stronger. However, it does not affect the effectiveness of the filtering algorithm. We use $Y_{I}$ to represent the peak-to-peak values of the I channel output signal, $Y_{Q}$ to represent the peak-to-peak values of the Q channel output signal, and $Y_{IQ}$ to represent the peak-to-peak values of the output signal of the algorithm. Then, $\eta =\frac{Y_{I}-Y_{IQ}}{Y_{I}}$ can be used to represent the noise-filtering performance of the algorithm. Figure 5 illustrates the noise-filtering performance of our algorithm under different acoustic frequencies and different angular velocities.

It was found that the orthogonal demodulation algorithm can effectively filter out acoustic interference signals of various frequencies. For example, when the angular velocity of the MEMS gyroscope is 0°/s and the sound frequency is 5592 Hz, the acoustic wave produces an interference of 0.5521 V in the output signal. After using the IQ dual-channel orthogonal demodulation algorithm, the acoustic interference signal was reduced to 0.0182 V, and the filtering performance reached as high as 96%. As shown in Figure 5, the filtering performance of the algorithm reached more than 95% in the theoretical simulation.

## 5. Experimental Verification of Noise Suppression Scheme Based on Orthogonal Demodulation

**Figure 6:**
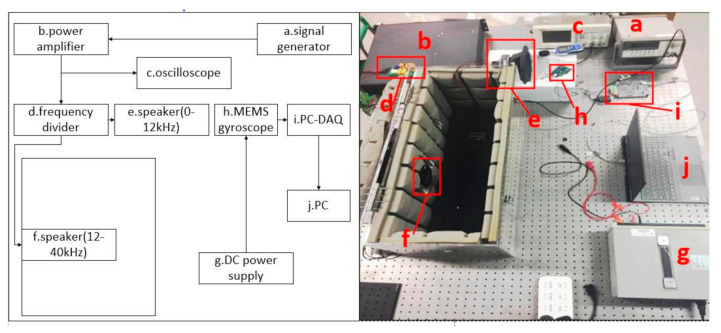
Structure of experimental equipment.

We set up an experimental platform, as shown in Figure 6, to prove the reliability of the filtering algorithm [13], and the acoustic interference experiment of the MEMS gyroscope prototype was carried out. The MEMS gyroscope was powered by a DC power supply GPS-2303C. The signal generated by the waveform generator Agilent 33250A was amplified by the power amplifier AE- TECHRON 7724 and transmitted to the speaker SV220WR-66-134-036 to produce high-frequency sound with a high sound pressure level. The output signal of the power amplifier was divided into two channels by the frequency divider. The sound frequencies between the two loudspeakers were different. We measured the response of the gyroscope by sweeping the sound signal, with the highest sweeping frequency reaching 40 kHz. A RIGOL DS1102E oscilloscope was used to observe the output signal of the power amplifier. The sound level meter HT-850A was used to measure the sound pressure level near the MEMS gyroscope. When the signal demodulated at a certain acoustic frequency was relatively flat, and the low-frequency signal was demodulated at a sound frequency greater or less than the previous acoustic frequency, it was determined that the driving frequency was approximately equal to the previous acoustic frequency. The measured driving frequency was approximately 6449 Hz. An acoustic wave with a sound pressure level of about 90 dB was used to produce interference and different acoustic frequencies were used for each data acquisition. High-frequency sampling of the MEMS gyroscope signal was achieved by using the data acquisition card USB6202. The sampling rate was set to 100 K, and the driving and sensing signals were obtained, which were imported into the noise elimination algorithm based on orthogonal demodulation as input. The collected voltage signal contained direct flow, which was removed during data processing.

**Figure 7:**
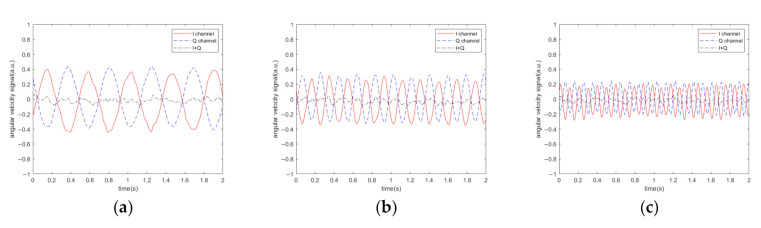
Demodulation output of the MEMS gyroscope at different acoustic frequency. (**a**) 6451 Hz; (**b**) 6454 Hz; (**c**) 6459 Hz.

**Figure 8:**
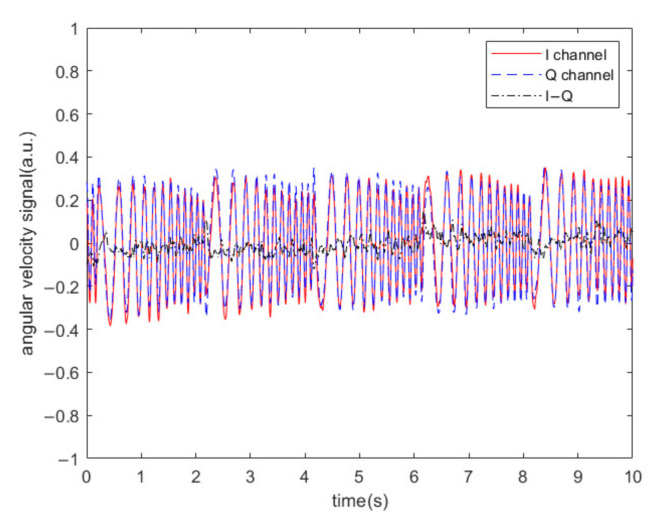
Demodulation output of the MEMS gyroscope when the sound signal is a sweep signal in the frequency range of 6451–6459 Hz.

Figure 7 shows the denoising effect when the sound frequency was greater than the driving frequency. The denoising effect is illustrated in Figure 8 when the sound signal was a sweep signal in the frequency range of 6451–6459 Hz, and the sweep period was 2 s. According to the algorithm, when the acoustic frequency is greater than the driving frequency, the two IQ channels are subtracted from each other to reduce the impact of noise. In this experiment, we did not apply an angular velocity to the MEMS gyroscope, so we removed the DC from the original demodulated signal.

**Figure 9:**
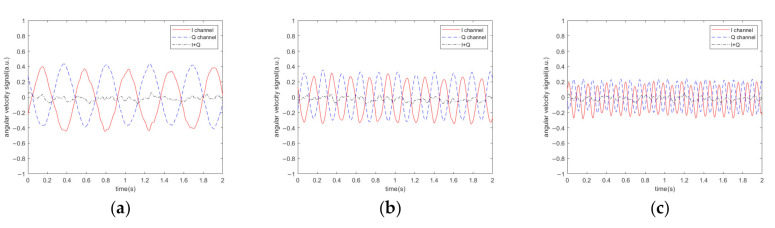
Demodulation output of MEMS gyroscope at different acoustic frequencies. (**a**) 6447 Hz; (**b**) 6444 Hz; (**c**) 6439 Hz.

**Figure 10:**
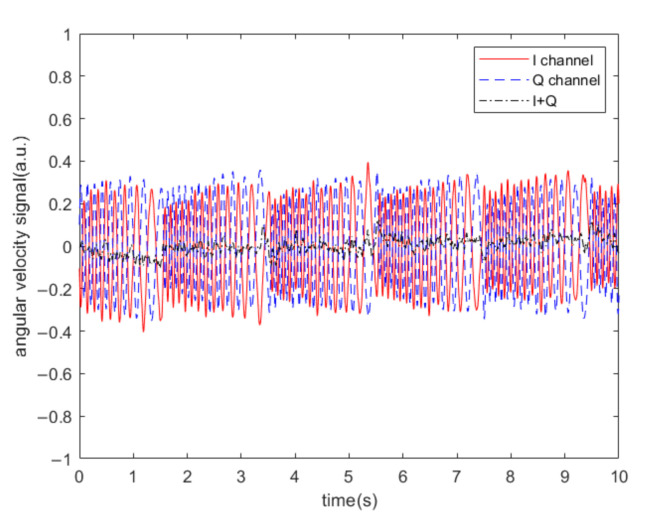
Demodulation output of the MEMS gyroscope when the sound signal is a sweep signal in the frequency range of 6439–6447 Hz.

According to the algorithm, when the acoustic frequency is less than the driving frequency, the two IQ channels can be added together to reduce the impact of noise. The corresponding denoising effect is shown in Figure 9. The denoising effect is illustrated in Figure 10 when the sound signal was a sweep signal in the frequency range of 6439–6447 Hz, and the sweep period was 2 s.

**Figure 11:**
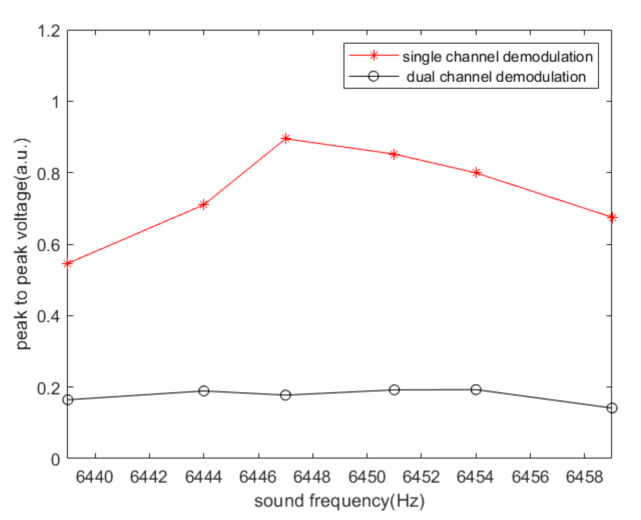
Experimental results of the peak-to-peak values of output signals when the angular velocity is 0°/s.

**Figure 12:**
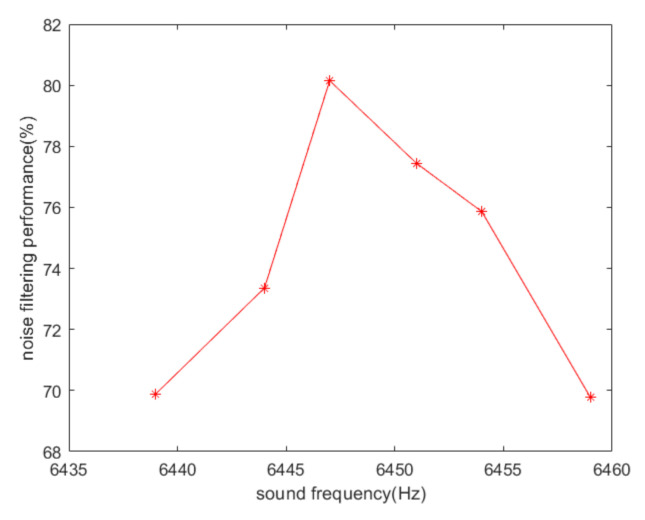
Noise-filtering performance of the MEMS gyroscope based on experimental data.

On the basis of the experimental data, the peak-to-peak values of output signals of single-channel and dual-channel demodulations under different sound frequencies are shown in Figure 11. Figure 12 illustrates the noise-filtering performance of our algorithm under different acoustic frequencies.

It can be seen from Figure 7, Figure 8, Figure 9, Figure 10 and Figure 11 that when the acoustic frequency is close to the driving frequency and the sound pressure level is sufficiently high, low pass filtering does not eliminate the influence of noise, the original single-channel demodulation scheme outputs the difference frequencies signal between the sound and the driving frequencies. Due to the resonance effect, the acoustic signal that is closer to the driving frequency has a greater impact on the demodulation output. The proposed algorithm can effectively filter out the difference frequency signal.

From the figure above, we can see that the noise-filtering performance in the simulation is much higher than that in the experiment. In fact, the output of the MEMS gyroscope in the experiment is not only interfered by acoustic interference. The experimental results show a larger peak-to-peak value after dual demodulation, and this is rarely caused by sound waves. Due to the manufacturing process defects of the gyroscope, the demodulation output is not completely flat even without acoustic interference and the angular velocity signal. Our scheme eliminates the interference signal caused by the sound wave acting on the sensing direction, which constitutes a large part of the overall interference. From the simulation results in Figure 5, it can be seen that the closer the sound frequency is to the driving frequency, the lower the noise-filtering performance obtained. The noise-filtering performance is reduced by no more than 3%. As for the experimental results in Figure 12, the loss in the noise-filtering performance is negligible because the greater impact of sound signal compensates for it. As the base noise is considered to be constant in a certain range, the closer the frequency of sound signal is to that of driving signal, the more effective our filtering scheme is.

## 6. Discussion

**Figure 13:**
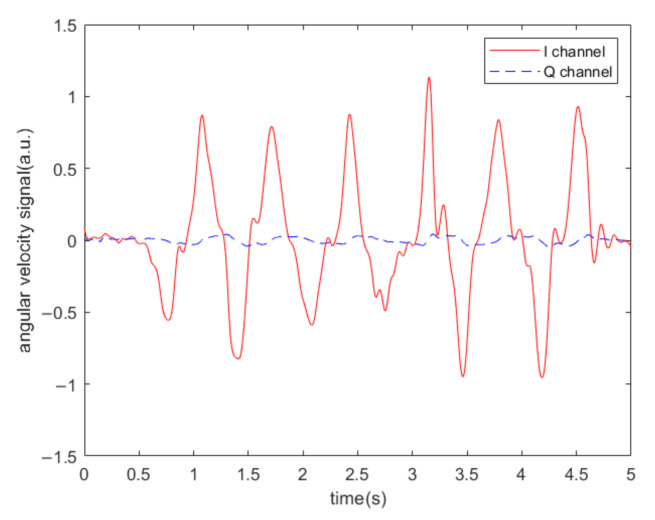
Output results of I channel and Q channel when random angular velocity is applied to the gyroscope without acoustic interference.

It should be noted that the precondition of the above experimental results is that the angular velocity of the gyroscope is 0°/s. In order to demonstrate that the algorithm does not filter out the angular velocity signal itself, we applied a random angular velocity signal without acoustic interference and collected the driving and sensing signals, and then used the scheme to perform the calculations. The output results of channel I and channel Q are shown in Figure 13. It can be seen that when the I channel output is the angular velocity signal, the Q channel output is close to 0. The algorithm does not affect the integrity of the angular velocity signal of the MEMS gyroscope.

**Figure 14:**
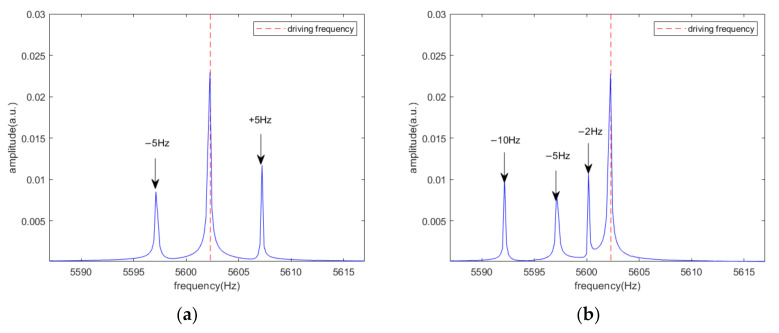
Frequency domain results. (**a**) Angular velocity signal; (**b**) Acoustic interference signal of three different frequencies.

**Figure 15:**
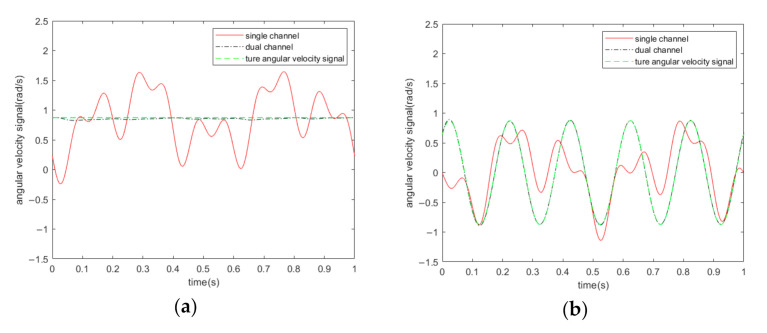
Output results of single-channel and dual-channel demodulations under acoustic interference of three different frequencies. (**a**) Unchanged angular velocity signal; (**b**) Angular velocity signal with an angular frequency of 5 Hz.

Figure 13 demonstrates the output of I channel and Q channel in the time domain, which shows that the output of Q channel is always 0 without acoustic interference. The angular velocity to be measured in Table 1 was set as the DC value. In reality, the angular velocity information may change with time. We assumed that $\Omega \left(t\right)=\Omega_{DC}\cos(2\pi ft)$, in which f = 5 Hz, the angular velocity to be measured is set to 50°/s, the angular velocity signal is an amplitude modulation signal of the bilateral band, as illustrated in Figure 14a, while Figure 14b shows the frequency domain results with acoustic interference of three different frequencies. The three frequencies are, sequentially, 2, 5, and 10 Hz away from the driving frequency. A comparison of Figure 14a with 14b shows that it is difficult for the sound signal to achieve the form in Figure 14a. In the case of acoustic interference, if the sound signal is a power spectrum with a continuous frequency variation within a given range, the proposed scheme can still suppress the side-band noise caused by sound. Under the triple-frequency acoustic attack shown in Figure 14b, the output of the single-channel and dual-channel demodulation schemes are shown in Figure 15. In order to provide a better comparison between the true angular velocity signal and the output of the algorithm, the direct current caused by the non-proportional damping coefficient $D_{yx}$ was removed in the simulation. It can be seen intuitively that the noise-suppression performance of the scheme was maintained.

Because the driving frequency of the gyroscope can reach kilohertz, it is difficult to achieve this kind of high-frequency noise in nature. It is relatively simple to realize in the laboratory and requires the use of specific devices. As introduced in Section 3, when the acoustic frequency is less than the driving frequency, by adding the two IQ signals, the noise can be suppressed. Otherwise, the two IQ signals should be subtracted. When acoustic signals are intentionally generated, whose frequencies change randomly above and below the driving frequency in the external environment, although rare, addition and subtraction between the I channel and the Q channel should be carried out in the algorithm. The results of the addition and subtraction are equal in length and can be divided into smaller time periods. For the data of both, the energy value of the signal in each small time periods should be calculated. When the acoustic frequency is close to the driving frequency, the demodulation output fluctuates, which means that the energy of the output data increases. As shown in Figure 15, the proposed scheme can greatly suppress the fluctuation and make the output stable. Therefore, whether IQ should be added or subtracted should be judged by the energy of each small time period. The data with the smaller energy value in each time period is taken as the output of the demodulation algorithm.

**Figure 16:**
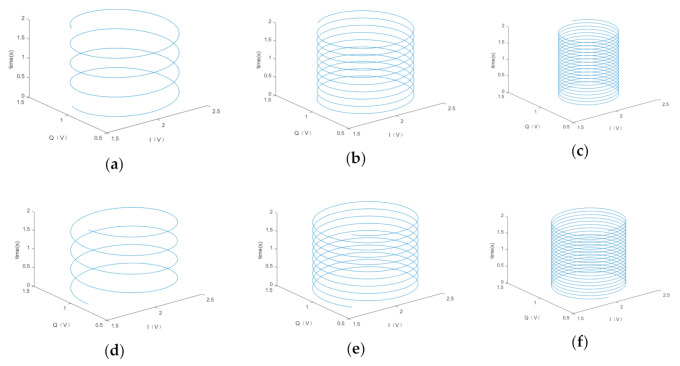
Simulation results of $V_{I}$ and $V_{Q}$ changing with time at different acoustic frequencies. (**a**) 5600 Hz; (**b**) 5597 Hz; (**c**) 5592 Hz; (**d**) 5604 Hz; (**e**) 5607 Hz; (**f**) 5612 Hz.

Compared with the traditional IQ demodulation scheme, our method adds a Hilbert transform to the Q channel. After the Hilbert transform, the effects of acoustic waves in the Q channel and I channel become components with the same frequency and phase, which is the last term of Equations (3) and (4). Therefore, the two-way difference can eliminate the acoustic interference. If the Q channel does not pass the Hilbert transform, the data of I channel and Q channel after low-pass filtering can be taken. They can be used to judge whether there is acoustic interference to realize early warning. When there is a difference between sound frequency and driving frequency, the variation of $V_{I}$ and $V_{Q}$ as a function of time are shown in Figure 16.

**Figure 17:**
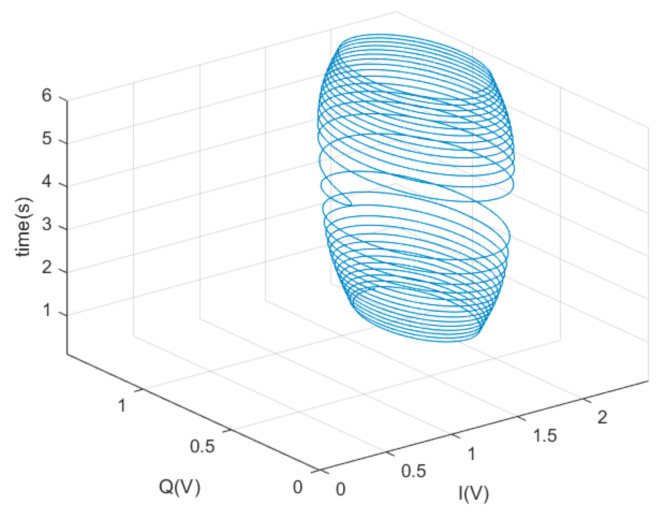
Simulation results of $V_{I}$ and $V_{Q}$ with linear sweep of sound signal over time.

Figure 16a–c shows the results when the acoustic frequency is less than the driving frequency, and Figure 16d–f shows the results when the acoustic frequency is greater than the driving frequency. In the case of acoustic interference, the demodulated output of I channel and Q channel changes with time in the form of a helix. From the time axis, the greater the difference between the acoustic and driving frequencies, the greater the frequency of the signal after demodulation, and the faster the helix rotates. Observing from the IQ plane, the direction of helix rotation depends on whether the acoustic frequency is greater or less than the driving frequency; the radius of the helix is reduced accordingly when the acoustic frequency is away from the driving frequency, which means that the acoustic interference is weakened. In the absence of acoustic interference, the outputs of I and Q channels are in the center of the helix. We can judge whether there is acoustic interference by observing whether or not a helix appears. Figure 17 illustrates the output of $V_{I}$ and $V_{Q}$ with a linear sweep of the sound signal over time. The time range was 0–6 s, and the sweep frequency changed from 5592 to 5612 Hz, and the driving frequency was in the center.

**Figure 18:**
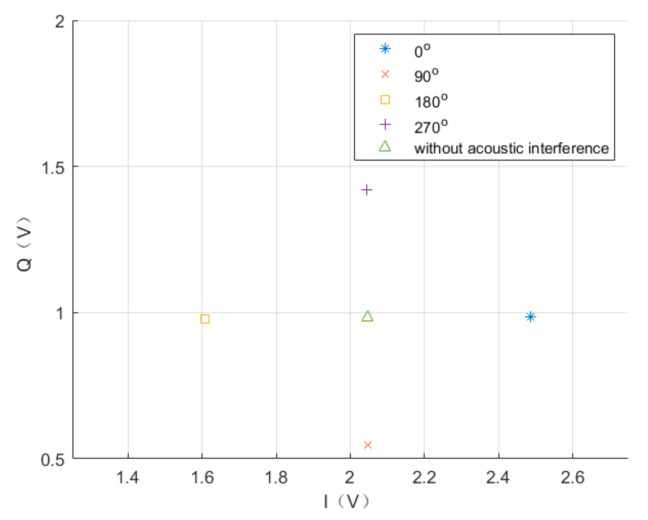
Output results of I channel and Q channel when the sound and driving signals had the same frequency and different phases.

It can be seen in Figure 17 that when the acoustic frequency reaches the driving frequency, the radius of the helix reaches the maximum value. At the same time, from the time axis, the demodulated signal frequency of I channel and Q channel is 0 Hz, and therefore it does not form a helix, but a straight line. The specific position of the straight line in space is determined by the phase difference between the sound and driving signals, as shown in Figure 18. The output of the I channel is maximal when the sound and driving signals have the same frequency and phase, while the minimum output is obtained when the phase difference is 180 degrees.

Although it is difficult to accurately control the acoustic frequency to stabilize it at the driving frequency in practical applications, if the acoustic frequency is equal to the driving frequency, the driving frequency can be slightly changed using other technical methods. As shown in Figure 16 and Figure 17, if the helix can be observed, it proves that there is acoustic interference at that time. The relevant personnel using MEMS gyroscopes can judge whether the gyroscope is disturbed by acoustic waves according to the difference between I channel and Q channel demodulation output signals, so as to make corresponding early warning and protection measures.

## 7. Conclusions

In this paper, by analyzing the working principle and failure mechanism of a MEMS gyroscope in the acoustic environment, the difference between the acoustic interference and angular velocity signals was compared. According to the original demodulation filtering algorithm, an IQ dual-channel orthogonal demodulation algorithm was proposed. From the theoretical and simulation analysis, this algorithm can eliminate the majority of the acoustic interference signal, with the filtering performance reaching 95%. Furthermore, we assembled the corresponding acoustic interference experimental system. The driving and sensing signals under acoustic interference were collected, and the reliability of the algorithm was verified. An acoustic warning scheme based on orthogonal demodulation was also proposed. This provides an algorithm and theoretical basis for the research of MEMS gyroscopes with anti-acoustic interference. In further work, we plan to fix the gyroscope prototype onto a rotating platform, apply constant angular velocity to the gyroscope, calibrate the angular velocity, and test the noise-filtering performance of the algorithm when a constant angular velocity is applied to the gyroscope.

## Figures and Tables

**Table 1:** Structural and acoustic parameters of MEMS gyroscope.

Parameter Name (Unit)	Symbol	Parameter Value
Driving mass (kg)	$m_{x}$	4 × 10^−9^
Sensing mass (kg)	$m_{y}$	2.5 × 10^−9^
Driving response frequency (rad/s)	$\omega_{x}$	2π × 5600
Sensing response frequency (rad/s)	$\omega_{y}$	2π × 5700
Driving damping coefficient(N·s/m)	$D_{xx}$	7.0372 × 10^−7^
Sensing damping coefficient(N·s/m)	$D_{yy}$	2.9845 × 10^−7^
Driving elastic coefficient(N/m)	$k_{xx}$	4.9522
Sensing elastic coefficient(N/m)	$k_{yy}$	3.2066
Non proportional damping coefficient(N·s/m)	$D_{yx}$	4 × 10^−9^
Unequal elastic coefficient(N/m)	$k_{yx}$	2.8159 × 10^−4^
Driving force(N)	$F_{x}$	1 × 10^−6^
Driving frequency(rad/s)	$\omega_{d}$	2π × 5602
Sensing equivalent sound pressure (N)	$F_{Ny}$	5 × 10^−9^
Noise frequency(rad/s)	$\omega_{n}$	2π × 5592, 2π × 5597, 2π × 5600, 2π × 5604, 2π × 5607, 2π × 5612
Angular velocity to be measured (rad)	$\omega_{t}$	2π × 0/360, 2π × 50/360, 2π × 90/360
